# Supplementary material for: What does mitogenomics tell us about the evolutionary history of the Drosophila buzzatii cluster (repleta group)?
Source: PLoS One. 2019 Nov 7;14(11):e0220676. doi: 10.1371/journal.pone.0220676 (PMC6837510; doi:10.1371/journal.pone.0220676)
Supplement: S1 Text — (DOCX) [file pone.0220676.s001.docx]

Set of primers used to sequenced each gene:

*COI*:

- (5’-TGAATTGCCTGATAAAAGGGTTACCTTGATAGGG-3')
- (5’-GCTCGTGTGTCTACGTCTATACCAACTGTAAA-3’)

*ND6*:

- (5’-GCGGCTACACCTAAAATTATATCTTAATCCAAC-3')
- (5’-TTAAAGAGTATAGATAAAATTGGTGCCAGCAATCGC-3’)

*rRNAL:*

- (5’-GGAGGAGCAGCTATATTAGCAGAACTTAA-3’)
- (5’-GGTGCAGGTAAATCAACTAAAGCATTATTAGC-3’)
