# Supplementary figures and images for: What does mitogenomics tell us about the evolutionary history of the Drosophila buzzatii cluster (repleta group)?
Source: PLoS One. 2019 Nov 7;14(11):e0220676. doi: 10.1371/journal.pone.0220676 (PMC6837510; doi:10.1371/journal.pone.0220676)

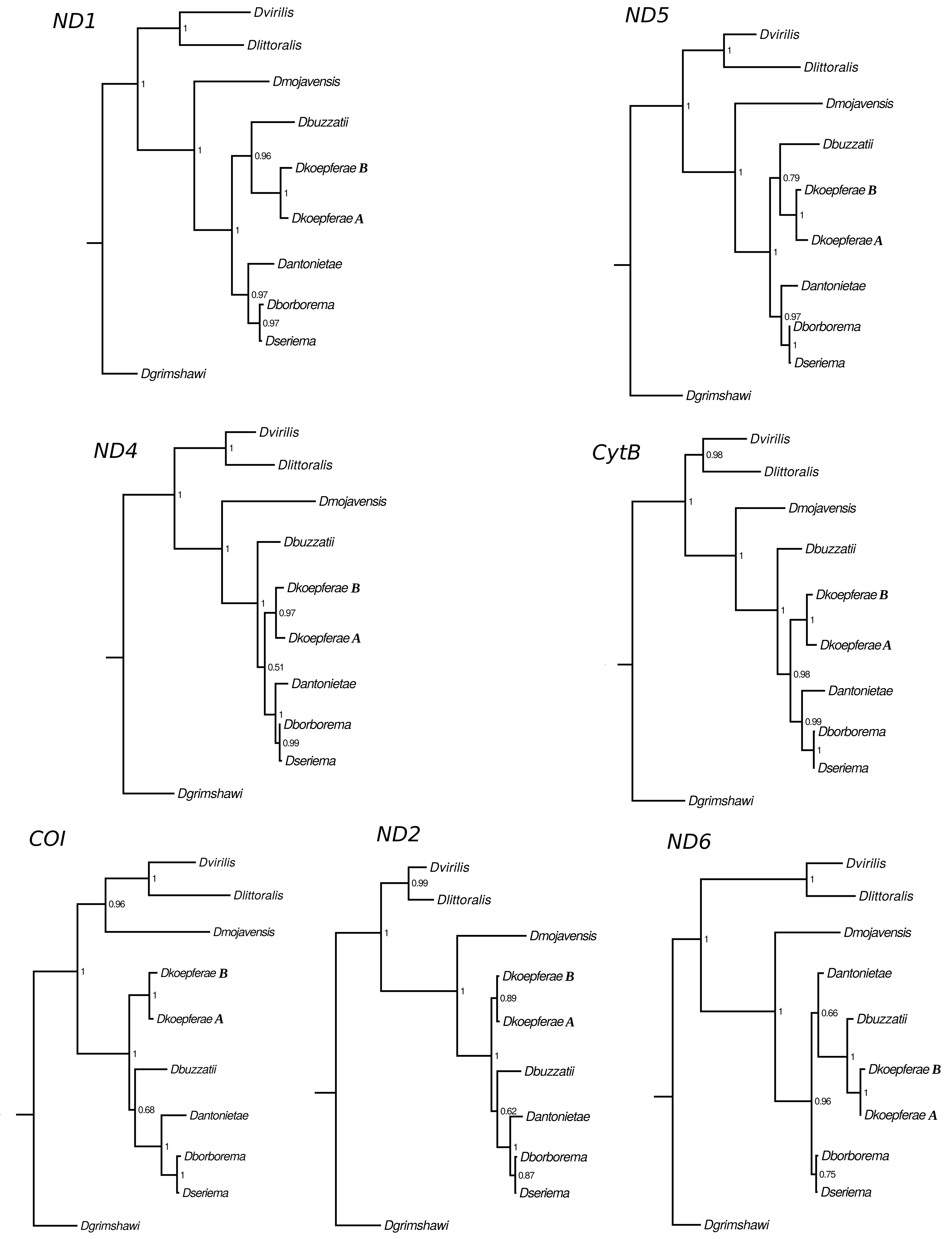

Supplement: S1 Fig — (TIF) [file pone.0220676.s004.tif]

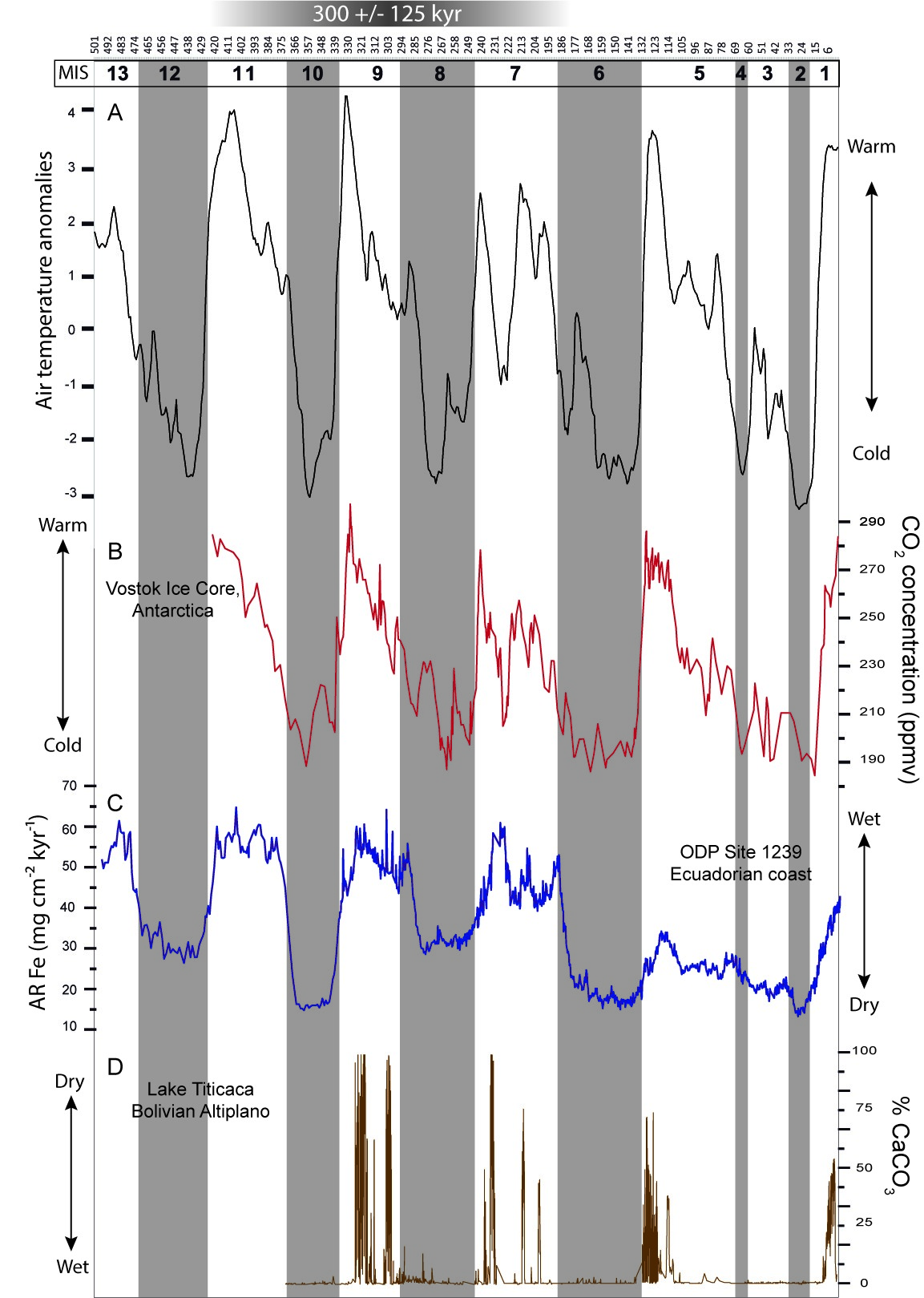

Supplement: S2 Fig — Ages in the top are indicated as 103 years (kyrs). Gradated shading area indicates divergence age estimates. Marine Isotope Stages (MIS) are labeled according to Lisiecki and Raymo [101]. Shaded vertical areas correspond to glacial periods whereas white areas correspond to interglacials or interstadials. Glacial periods correspond to cold and dry conditions in the western slopes of the Western Andes, and cold and wetter conditions in the eastern slopes of the Eastern Andes and the Altiplano. A. Globally-averaged surface air temperature anomaly reconstructed from proxy and model data for the last eight glacial cycles [108]. B. CO2 concentration based on Vostok Ice Core data [109]. C. Iron accumulation rates (AR Fe) reflecting changes in terrigenous sediment input to ODP Site 1239D, Equatorial Pacific [110]. D. % of CaCO3from Site LT01-2B indicating changes in water balance at Lake Titicaca Basin, Bolivia (modified from [106]). (TIF) [file pone.0220676.s005.tif]
