# Supplementary material for: What does mitogenomics tell us about the evolutionary history of the Drosophila buzzatii cluster (repleta group)?
Source: PLoS One. 2019 Nov 7;14(11):e0220676. doi: 10.1371/journal.pone.0220676 (PMC6837510; doi:10.1371/journal.pone.0220676)
Supplement: Attachment — Submitted filename: Response_to_Reviewers.docx [file pone.0220676.s007.docx]

Editorial Board

Dr. William J. Edges

Academic Editorial

Dear Dr. Edges,

First, we would like to thank the subject editor Dr. W.J. Etges and the two reviewers, an anonymous one and Dr. D. Rand, for helpful and insightful comments that have definitely contributed to improve manuscript entitled “What does mitogenomics tell us about the evolutionary history of the Drosophila buzzatii cluster (repleta group)?” by Nicolás Moreyra et al.

We revised the manuscript addressing all the issues raised by the academic editor and both reviewers. Below, we transcribe each one of the comments, followed by our response (Author´s response), which outlines the changes made to the manuscript in order to reflect the suggestions made in each case.

**Responses to the Academic Editor**

Two external referees and me have now evaluated your paper. Both reviewers raised some very important issues that need to be addressed in a revision. I re-read the MS and found a large number of grammar and translation issues that must be corrected satisfactorialy in a revised MS. I have uploaded a marked up manuscript to aid you in the revision process. I have made many comments that need to be addressed in this marked up manuscript, but I may have missed some that need fixing. Please proofread carefully.

**Author’s response:** all comments and suggestions in the manuscript marked up by the editor were taken into account and incorporated in the revised version.

A few other points need explanation, such as why some in-group species were not included in this analysis, and some cautionary explanation about the use of single inbred lines to represent these species, except for D. koepferae. I'd guess that more geographically disparate samples would complicate this analysis and change your conclusions as well.

**Author’s response:** *D. gouveai* and *D. serido* are species that inhabit Brazilian arid lands, and could not be included in this study because of the unavailability of stocks in *Drosophila* repositories for these species. This is detailed in the revised manuscript. We included a sentence in the discussion section providing a cautionary note related to the use of single lines as representative of species, except for Dkoe, which was represented by two highly differentiated lines.

Further, using a "total evidence" approach, why not use all available genetic data including inversions to build these trees? I don't think its surprising that mtDNA yields different results than nuclear markers. However, the acquisition of more mitogenomes for these species is a valuable exercise.

**Author’s response:** We did not included other genetic data to build the trees because in a recent phylogenetic study based on a large nuclear genes dataset, considered other sources of information, like morphology and inversions. The aim of the present study was to compare the results of our mitogenomic approach with the study based on transcriptomic data.

**Response to Journal Requirements**

2. In your Methods section, please provide additional location information of the collection sites, including geographic coordinates for the data set if available.

**Author’s response:** more information about location and collection of species was added in the manuscript.

3. In your Methods section, please provide additional information regarding the permits you obtained for the work. Please ensure you have included the full name of the authority that approved the field site access and, if no permits were required, a brief statement explaining why.

**Author’s response:** Two of the stocks, Dbrb and Dkoe B derived from collections performed by other long time ago (see materials and methods section for details). The mitogenome of *Dsei* was assembled using reads obtained in G. Kuhn's lab. Dbuz, Dkoe A and Dato derived from collections performed by members of our lab in 2010.

4. We note that Figure 1 in your submission contain map/satellite images which may be copyrighted. All PLOS content is published under the Creative Commons Attribution License (CC BY 4.0), which means that the manuscript, images, and Supporting Information files will be freely available online, and any third party is permitted to access, download, copy, distribute, and use these materials in any way, even commercially, with proper attribution. For these reasons, we cannot publish previously copyrighted maps or satellite images created using proprietary data, such as Google software (Google Maps, Street View, and Earth). For more information, see our copyright guidelines: [http://journals.plos.org/](http://journals.plos.org/plosone/s/licenses-and-copyright).

**Author’s response:** the map image shown in Figure 1 was prepared using SimpleMappr map website [[https://www.simplemappr.net](https://www.simplemappr.net/)], that contain data in Public Domain for modifying, distributing and printing. The primary author, David P. Shorthose has waived all copyrights. We included an statement in the legend of Figure 1 and we attached a document “Other.docx” where we add more information.

6. We note that you have included the phrase “data not shown” in your manuscript. Unfortunately, this does not meet our data sharing requirements. PLOS does not permit references to inaccessible data. We require that authors provide all relevant data within the paper, Supporting Information files, or in an acceptable, public repository. Please add a citation to support this phrase or upload the data that corresponds with these findings to a stable repository (such as Figshare or Dryad) and provide and URLs, DOIs, or accession numbers that may be used to access these data. Or, if the data are not a core part of the research being presented in your study, we ask that you remove the phrase that refers to these data.

**Author’s response:** we decided to remove the phrase that refers to this result.

**Responses to reviewers**

**Reviewer #1:**

Specific suggestions.
Please, when necessary (e.g. Figure 3) use the official FlyBase abbreviations for Drosophila species names.
Dato = D. antonietae
Dbrb = D. borborema
Dbuz = D. buzzatii
Dkoe = D. koepferae
Dsei = D. seriema

**Author’s response:** we adopted the official FlyBase abbreviations for the species names.

To refer to the two strains of D. koepferae you should use always the same name: either A and B, or 11 and 7.1. Currently there is a mixture, compare Figure 3 and Figure 4.

**Author’s response:** we corrected references to strains of *D. koepferae* to A and B (instead of 11 and 7.1) in the text, Figure 3, Figure 4 and Figure 5 (as well as in S1 Fig).

Page 2, lines 29-30. I suggest to use the alphabetical order for species names (as in Table 1): D. antonietae, D. borborema, D, buzzatii, D. koepferae (two stocks) and D. seriema.
Figure 3. I don’t see the logical in the order of the pairwise comparisons. Perhaps you could use the alphabetical order (in agreement with previous comment):

Dato – Dbrb
Dato – Dbuz
Dato – Dkoe A
Dato – Dkoe B
Dato – Dsei
Dbrb – Dbuz
Dbrb – Dkoe A
Dbrb – Dkoe B
Dbrb – Dsei
Dbuz – Dkoe A
Dbuz – Dkoe B
Dbuz – Dsei
Dkoe A – Dkoe B
Dkoe A – Dsei
Dkoe B – Dsei

**Author’s response:** We change the order of the species names in Table 1 to alphabetical order. However, we included *D. mojavensis* in the last column (avoiding the sorted list) as a comparative external reference.

Pages 6-7, lines 144-155. It is not clear to me whether the authors produced a “de novo” assembly of the six mitogenomes or they used D. mojavensis as a template. This is important in order to conclude (page 10) that PCGs in the six mitogenomes show the same order and orientation than in D. mojavensis. Please clarify this issue.

**Author’s response:** We produced a reference assembly only for the conserved regions between *D. mojavensis* and each species belonging the *buzzatii* cluster, creating several templates (based on different subsets of reads) for each species. After that, the complete set of mitochondrial reads (of each species) was mapped to the corresponding templates to generate several genome assemblies that were aligned to create a consensus per species. We also performed de novo assemblies for each species (line 166) that were more fragmented, but happily they revealed the same gene order along the mitogenomes. These details are clarified in the revised manuscript.

Page 11, lines 246-247. Please give more details on how pairwise nucleotide diversity (pi) was calculated. Because you have six mitogenomes in the buzzatii cluster, there is a total of 15 pairwise comparisons. Is the value reported in Figure 2 the average of the 15 pairwise comparisons? If this is the case, this value includes both interspecific and intraspecific (two stocks of *D. koepferae*) comparisons. Clarify.

**Author’s response:** we estimated nucleotide divergence by calculating the p-distance (analogous of ***pi***), which is obtained by dividing the number of nucleotide differences between species pairs by the total number of nucleotides compared and by the number of pairs of sequences being compared (six in our study). We decided to independently include stocks of *D. koepferae* given the significant divergence between them, which is even greater than between *D. borborema* and *D. seriema*. In order to avoid misunderstandings regarding the parameter *pi* we decided to refers the measure of divergence as p-distance (instead of *pi* since this parameter usually refers to polymorphisms within populations).

Minor corrections
Page 2, line 33. The monophyly of that the D. buzzatii cluster. Delete “that”
Page 5, line 108. Insert “cluster” after buzzatii
Page 6, line 134. Split the sentence:
… unpublished data). For D. seriema and D. buzzatii, mitochondrial reads were retrieved …
Page 13, line 283. We could only obtain trees for ….
Page 13, line 287. Allocated -> placed
Page 13, line 294. Legend of Fig 4. Bootstrap values and posterior probabilities are indicated at each node.
Page 15, line 334. Thus -> on the other hand,
Heading of Table S1. Set of primers used to sequence each gene.

**Author’s response:** all corrections made

**Reviewer #2:**

Line 33. Delete extra ‘that’
**Author’s response:** corrected

Line 246: A terminology point: ‘pi’ for pairwise diversity usually refers to polymorphism within populations, and estimates this value from a random sample of alleles. The use of pi from a sample of species has a somewhat different interpretation given that taxon sampling is different from a random sample of alleles from a breeding population. The use of a pairwise metric is fine, but some clarification should be made in these different contexts.

**Author’s response:** see above in responses to reviewer 1.

Line 248: The pi values are larger in the melanogaster subgroup than in the buzzatii cluster. Related to the comment above, if these were equivalent random samples from two different idealized populations the difference would imply different population genetic forces, but since the differences in divergence times and distribution of taxa in the two subgroups / clusters being compared are not the same, the pi metric may simply reflect those historical contingencies. The metric pi is sensitive to the frequencies of alleles in a population, so more closely related mtDNAs will reduce pi, or more deeper-diverging mtDNAs will inflate it. So, some comment should be made to this effect to provide context for the larger pi values in the melanogaster subgroup. Notably, that group has three very diverged mtDNAs within the D. simulans clade that may inflate pi, which is not seen in the buzzatii sample. Clarify that the Dmel vs. Dbuzz difference is probably due to depth of divergence, not something distinct about rates of evolution. The use of a pairwise metric is fine, but its interpretation is complicated by the difference between a taxon sample vs. an allele sample from a breeding population.

**Author’s response:** we included a sentence explaining that the higher overall divergence observed for the *melanogaster* subgroup may be due to the deep divergence between some of the species pairs it includes, eg. *D. melanogaster* and *D. yakuba*, that diverged more than 10 MYA.

Lines 261-263: The higher dN/dS values for two genes in Dbuzz vs. Dmel data (Table2), may imply a shift in function. It could be noted that since the dS values (and pi as per points above) is higher in Dmel group, the true value of dS may be slightly underestimated due to multiple hits, leading to a slight overestimation of the dN/dS ratio in Dmel. This means that the elevated values of dN/dS for the ND and ATP genes in the Dbuzz group relative to the same Dmel genes are probably real.

**Author’s response:** We added a sentence (line 276) addressing the point raised by reviewer 2.
